# Population Genetics of the Eastern Hellbender (*Cryptobranchus alleganiensis alleganiensis*) across Multiple Spatial Scales

**DOI:** 10.1371/journal.pone.0074180

**Published:** 2013-10-18

**Authors:** Shem D. Unger, Olin E. Rhodes, Trent M. Sutton, Rod N. Williams

**Affiliations:** 1 Savannah River Ecology Laboratory, University of Georgia, Aiken, South Carolina, United States of America; 2 School of Fisheries and Ocean Sciences, University of Alaska Fairbanks, Fairbanks, Alaska, United States of America; 3 Department of Forestry and Natural Resources, Purdue University, West Lafayette, Indiana, United States of America; George Washington University, United States of America

## Abstract

Conservation genetics is a powerful tool to assess the population structure of species and provides a framework for informing management of freshwater ecosystems. As lotic habitats become fragmented, the need to assess gene flow for species of conservation management becomes a priority. The eastern hellbender (*Cryptobranchus alleganiensis alleganiensis*) is a large, fully aquatic paedamorphic salamander. Many populations are experiencing declines throughout their geographic range, yet the genetic ramifications of these declines are currently unknown. To this end, we examined levels of genetic variation and genetic structure at both range-wide and drainage (hierarchical) scales. We collected 1,203 individuals from 77 rivers throughout nine states from June 2007 to August 2011. Levels of genetic diversity were relatively high among all sampling locations. We detected significant genetic structure across populations (F_st_ values ranged from 0.001 between rivers within a single watershed to 0.218 between states). We identified two genetically differentiated groups at the range-wide scale: 1) the Ohio River drainage and 2) the Tennessee River drainage. An analysis of molecular variance (AMOVA) based on landscape-scale sampling of basins within the Tennessee River drainage revealed the majority of genetic variation (∼94–98%) occurs within rivers. Eastern hellbenders show a strong pattern of isolation by stream distance (IBSD) at the drainage level. Understanding levels of genetic variation and differentiation at multiple spatial and biological scales will enable natural resource managers to make more informed decisions and plan effective conservation strategies for cryptic, lotic species.

## Introduction

Rivers are complex, dynamic systems that shape aquatic ecosystems at the landscape scale through a combination of biotic and abiotic processes. A central theme in stream conservation biology involves assessing spatial and temporal patterns of genetic variation within species inhabiting streams distributed across landscapes [1]. Conservation geneticists have developed a number of statistically rigorous tools for characterizing the genetic attributes of species inhabiting lotic ecosystems, including examining genetic diversity across multiple scales, and thus evolutionary potential [2]. Quantifying genetic variation and population connectivity across a dendritic arrangement of rivers can further our understanding of population specific evolutionary trajectories essential for effective conservation management of imperiled species within watersheds. As stream populations of aquatic species become increasingly isolated and fragmented they may exhibit reduced levels of genetic variation, ultimately leading to significant differentiation due to random genetic drift and increased risk for extinction [3]. The long-term viability of species and the maintenance of overall aquatic biodiversity rely on the degree to which riverscapes facilitate demographic and genetic exchange among populations [4], [5]. Bayesian clustering tools derived from the field of landscape genetics, or “riverscape genetics” [6], can be utilized to infer the numbers of populations that exist across the ranges of aquatic species [7], [8], as well as to resolve fine-scale patterns of genetic structure across basins, sub-basins, and stream reaches (individual streams) at the drainage level [9], [10], [11].

While numerous investigators have assessed the genetic diversity and structure of fish and macroinvertebrate species within and among lotic systems [12], [1], relatively few have investigated the genetic attributes and spatial connectivity of stream-dwelling amphibians [13], [11]. The lack of research on genetic and biological connectivity of stream dwelling amphibians is surprising given that many amphibian populations are experiencing declines worldwide due to increasing habitat fragmentation, spread of disease, increased UVB radiation, and habitat degradation [14], [15], [16]. As many as one third of the currently described amphibian species have undergone extinction or severe declines [17], with the most severe declines occurring within streams [18]. While various biological responses to habitat reduction for stream salamanders have been documented, levels of genetic diversity and characterization of gene flow at the watershed scale has rarely been quantified [19], [20]. Because of the linear nature of stream systems, many aquatic species exhibit strong correlations between genetic variance partitioning and drainage connectivity [21], [22], which allows for genetic approaches to decipher biological connectivity among populations of aquatic amphibian species over varying spatial scales.

The eastern hellbender *Cryptobranchus alleganiensis alleganiensis* is a large, long-lived, aquatic salamander which is confined to lotic dispersal. Hellbenders exhibit a strictly North American distribution, currently ranging from New York, across the Midwest to Missouri, and through several southern states to northern Georgia [23]. Hellbender populations are declining across their range, in some areas up to 77% [24], with declines attributed to increases in such factors as stream impoundment, siltation, gigging activities, scientific collection, illegal harvest, canoe traffic, agriculture runoff, predation by non-native fishes, and thermal pollution [23], [24], [25]. Many “at risk” hellbender populations are composed of older age classes with little to no signs of recruitment and have the potential for significant losses of genetic diversity due to small population sizes. It also is thought that isolated demes of hellbenders may be susceptible to the Allee effect [26], especially considering that individuals are often restricted to intra-river movements [24], [27], [28], [29]. Due to ongoing conservation concerns, it is imperative to elucidate the genetic consequences of these demographic declines observed in eastern hellbender populations.

Recent studies of eastern and Ozark hellbender *Cryptobranchus alleganiensis bishopi* phylogeography utilizing mtDNA and microsatellite markers, have divided the species range into eight reciprocally monophyletic groups with negligible gene flow among groups [30], [31]. Moreover, high genetic structure and differentiation between rivers within Missouri have recently been documented (F_st_ average = 0.40; [32]) for both eastern and Ozark hellbenders. However, while we now have more data with which to resolve the genetic landscape of hellbenders in North America, these previous studies lacked both comprehensive sampling efforts replicated across watersheds at multiple spatial scales as well as highly polymorphic, species-specific markers with which to evaluate patterns of genetic structure in this species. Unfortunately, the need for more precise resolution of the genetic and biological processes of North American hellbenders has never been more critical, as evidenced by the recent listing of the Ozark hellbender subspecies as federally endangered and the entry into candidate status for listing of the eastern subspecies (J. Applegate, personal communication).

To provide the resolution needed for making informed conservation and management decisions for eastern hellbenders, our goal in this research was to perform exhaustive sampling across the range of the eastern hellbender and to use these samples to detect genetic signatures of reduced population size (i.e., bottlenecks, inbreeding, decreases in heterozygosity), delineate genetically distinct populations, and provide baseline data for conservation efforts [33]. The primary objectives of this study were to 1) examine levels of genetic diversity and structure across the geographic range of the eastern hellbender, 2) to infer the number of subpopulations of eastern hellbenders range-wide and at the drainage scale using Bayesian clustering methods, 3) describe patterns of genetic isolation by distance at the drainage level, and 4) to examine the hierarchical partitioning of genetic variation in eastern hellbenders within dendritic stream networks. Specific outcomes of this research should enable conservation managers to define the range-wide genetic structure of eastern hellbenders and provide the empirical data needed to identify source populations for watershed specific hellbender population augmentation and translocation programs. This study has broad implications by providing a paradigm for the influence of basin architecture on a declining lotic species.

## Methods

### Ethics Statement

Permits to collect tissue samples included the Indiana Department of Natural Resources (#09-0161), North Carolina Division of Wildlife Resources (#NC-2010ES286), Pennsylvania Fish & Boat Commission (#019-755-578), Georgia Wildlife Resources Division (#29-WBH-10-184), Tennessee Wildlife Commission (#3564), National Park Service Great Smoky Mountains NP (#GRSM-2010-SCI-0031), and Purdue University Animal Care and Use committee (#UNG-895).

### Range-wide Sampling Design

**Figure 1:**
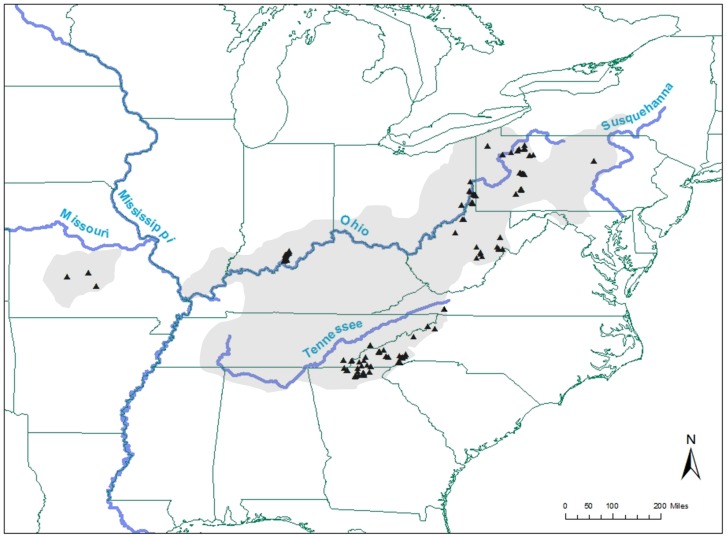
Sample locations and major drainages used in range-wide study of eastern hellbenders. Triangles represent sample locations within major drainages of the Ohio, Tennessee, Susquehanna, and Missouri Rivers. Range map (shaded grey) reprinted from [67] under a CCBY license, with permission from Petranka, original copyright 1998.

Eastern hellbenders were sampled across major watersheds throughout their current geographic range. We collected a minimum of 25–50 samples per watershed across several streams within major river basins of the Ohio, Tennessee, Mississippi, and Susquehanna River basins (Figure 1). Genetic samples for range-wide assessment were collected between June 2007 and August 2011 in 77 discrete rivers across nine states (Figure 1). Genetic samples consisted of either a small tail clip, ∼2–5 mm in size, stored in 95% ethanol or blood samples collected and preserved in lysis buffer (1 M Tris, 0.5 M EDTA pH 8.0, 5 M NaCl, 20% SDS; [34]). Upon capture of each salamander, we recorded sample locations as UTM coordinates as well as age class (adult, sub-adult, juvenile). While field sampling, technicians searched in an upstream direction and released individuals at their point of capture after processing to ensure the same individuals were not resampled in study areas where individuals were unmarked.

### Drainage Scale Sampling Design

**Figure 2:**
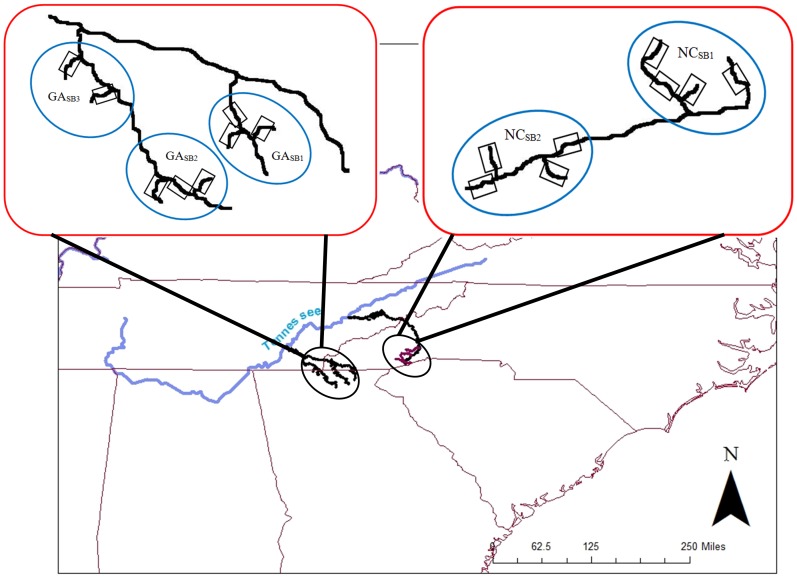
Drainage (Hierarchical) scale sample design for eastern hellbenders showing basin (red rounded box), sub-basins (blue circles), and stream reaches within sub-basins (boxes). All basins are within the Tennessee River drainage. Basins consist of French Broad River, North Carolina (sub-basins NC_SB1_, NC_SB2_) and Hiawassee River, Georgia (GA_SB1_ GA_SB2_, & GA_SB3_).

To assess hierarchical patterns of genetic structure across stream networks embedded within a specific drainage, we used those hellbender samples obtained within the Tennessee River drainage. This river drainage was chosen due to the presence of stable populations and ability to collect minimal sample sizes of 15–20 adults per stream reach. This drainage-scale study consisted of three hierarchal levels: basins, sub-basins, stream reaches (individual streams) and generally followed the sample design of Finn et al. 2007 [12] and Mullen et al. ([11]; Figure 2). We sampled within two major basins within the Tennessee River drainage: the French Broad River located in western North Carolina and the Hiawassee River in northern Georgia. Within these basins, we sampled multiple sub-basins, two within North Carolina (NC_SB1_ and NC_SB2_) and three within Georgia (GA_SB1_, GA_SB2_, and GA_SB3_; Figure 2). We sampled four stream reaches within NC_SB1_, four in NC_SB2_, three within GA_SB1_, two within GA_SB2_, and three within GA_SB3_. At least 15–20 individuals per stream reach were collected (with the exception of one stream within NC_SB1_; n = 13) to ensure sufficient power to detect genetic structure. This sampling regime allowed us to examine genetic variation at multiple hierarchical scales: within the Tennessee River Basin overall, within and between basins (Georgia and North Carolina), within and among sub-basins within basins (NC_SB1_ versus NC_SB2_, GA_SB1_ versus GA_SB2_ versus GA_SB3_), and within and among stream reaches nested within sub-basins.

### Laboratory/Quality Control

Genomic DNA was extracted from all samples using a standard phenol-chloroform protocol [35], [36]. Individuals were multiplexed across 12 microsatellite markers following the thermal profiles described in Unger et al. 2012 [34]. The PCR products were analyzed on an ABI 3739XL automatic sequencer and genotyped using GENEMAPPER version 3.7. Quality-control measures followed Unger et al. 2012 [34] and included re-amplification of genotypes with low signal intensity, independent scoring of a random subset (10%) of our data to identify genotyping errors and reamplification and scoring of approximately 20% of all genotypes. GENALEX 6.41 [37] was used to screen for any redundant genotypes.

## Analysis

### Range-wide Scale

#### Genetic Variation

We estimated standard metrics of genetic diversity for each stream reach including allelic richness (A), number of private alleles (A_p_), observed (H_o_), and expected heterozygosites (H_e_) across all loci in GENEPOP [38]. Deviations from Hardy-Weinberg (HWE) equilibrium within each stream reach (population) were calculated in GENALEX [37]. The frequency of null alleles was estimated with MICROCHECKER [39] for the overall data set. Weir and Cockerham's [40] estimate of F_is_ values were calculated and tested for significant deviations from Hardy-Weinburg equilibrium (HWE) averaged across populations in FSTAT [41].

#### Population Genetic Structure

To assess levels of differentiation and population structure between streams at the range-wide scale, we first calculated Fst values between all pair-wise combinations of stream reach (population) using 1,000 randomizations in FSTAT (θ; [40], [41]. Eastern hellbender populations characterized by limited gene flow and high site philopatry should result in detectable levels of population structure and a distinct pattern of differentiation among stream reaches (populations). Due to the large number of alleles per locus in our database, we ran an additional measure of genetic differentiation, Jost'D [42] using SMOGD [43].

Our second evaluation of range-wide genetic structure utilized the Bayesian clustering method STRUCTURE to assign individuals to genetic populations or clusters (*K*) based on the minimization of Hardy Weinberg and linkage disquilibrium (HWE) within clusters [44], [8], [45]. In STRUCTURE, multiple preliminary runs were performed to evaluate the potential support for varying numbers of populations (*K, 1–100*) in the dataset. The maximum *K* of 100 was set to account for the potentially large number of clusters that might exist among the 77 distinct rivers. Our maximum *K* was adjusted to 10 after determining the highest likelihood values for *K* were under 10. To infer the actual number of clusters supported by our dataset, we used the Δ*K* method of Evanno et al. 2005 [46] in STRUCTURE HARVESTER [47]. We also determined the plateau of likelihood value plots for each value of *K* and qualitatively examined increases in variance after the “true” *K* was reached according to Pritchard et al. [48]. To assign individuals to subpopulations, we performed a total of ten runs (*K* = 1 to10) as well as a final run at the inferred *K* (*K* = 2), consisting of 1,000,000 iterations after a burn in of 100,000 to ensure stabilization of our MCMC (Markov chain Monte Carlo; which we confirmed with additional runs of 2 million iterations and 200,000 burn in yielding the same result). STRUCTURE was run under the uncorrelated alleles model to prevent overestimation of *K* given our sample design (high clumping of samples within streams; [45], [49]. We used the admixture model, as it is more robust for potential inclusion of admixed individuals and detecting fine scale population structure for weakly differentiated clusters [50]. We utilized STRUCTURE HARVESTER to graphically visualize the number of *K*'s, log likelihood values, and variance of STRUCTURE output to infer the number of clusters using multiple methods [47].

In STRUCTURE we averaged q values, the proportion of an individual's sampled genome characteristic to each subpopulation over all runs. We then confirmed assignment of individuals to specific groups using the cutoff of 70% assignment as per Latch et al. 2008 [51]. The run with the highest log-likelihood value for a given *K* was used to assign q values to individuals and plotted the results on a map to assess geographical congruence.

### Drainage Scale

To assess levels of genetic divergence at the drainage level, pairwise F_st_ values from eastern hellbenders sampled from streams within the Tennessee River drainage were performed in FSTAT [41]. In addition, hierarchical analyses of molecular variance (AMOVA) were performed in ARLEQUIN [52] to quantify the partitioning of genetic variance within and among the hierarchal levels of this drainage (i.e., basin, sub-basin, and stream reach). In total, three AMOVAs were performed, one within each basin (i.e., North Carolina and Georgia) and one among both basins (global; [53]). Genetic structure was evaluated at three hierarchical levels within each basin: within and among sub-basins within basins, within and among stream reaches within sub-basins, and within stream reaches. Hierarchical *F* statistics for this analysis consisted of *F*_b_ (divergence among basins), *F*_sb_ (divergence among sub-basins within basins), *F*_srsb_ (divergence among stream reaches within sub-basins), and *F*_is_ (inbreeding coefficient of stream reaches; [53], [12]). We ran a Principle Components Analysis (PCA) on allele frequencies across all 12 loci within streams to visually assess genetic structure at the drainage scale in PCORD [54]. This ordination approach provides a direct comparison of allelic distribution across the watershed drainage versus traditional F statistics or Jost's D [55].

To investigate levels of philopatry and vagility of eastern hellbenders within individual streams, we tested for isolation by stream distance (IBSD) at the drainage scale. For this analysis the linear stream distance between sample locations in kilometers was compared to stream F_st_ values and tested for correlation using linear regression analysis. Linear stream distance was measured in ARCMAP 10.1 (Environmental Systems Research Institute Inc). GPS coordinates of individual captures were used to determine geographic distance between individual sample locations. This analysis provides an additional measure of genetic distance to describe the relationship between geographic and genetic distance at the scale appropriate for hellbender life history.

To test whether genetic structure reflected the topography of streams and identified stream sections that contributed the most to genetic differentiation, we used STREAMTREE [56]. North Carolina sub-basins were used as they conformed to a spatial arrangement consistent with this approach by having well defined tributaries connected by the same mainstem. This analysis infers the relative genetic distance between sample locations along stream sections based on a matrix of pairwise F_st_ values. STREAMTREE allowed us to qualitatively compare results from the ISBD analysis. This software determines a coefficient of determination (R^2^) to infer fit of the data to the STREAMTREE model of stream hierarchy in which gene flow is confined to a one-dimensional space utilizing watershed specific topology to aid in identification of stream barriers in the absence of strong isolation by distance pattern [57].

To infer the number of distinct genetic clusters at the drainage scale, we used STRUCTURE (which was run under similar parameters as the range-wide scale) and GENELAND [58]. GENELAND can incorporate a spatial component by using geographic data to inform the clustering of individuals into populations and is appropriate for analysis of population structure at this scale (within drainages; [58]). In GENELAND, minimum and maximum values for *K* were set similar to STRUCTURE; initially 1–10. The poisson maximum was set to 360, while the Poisson-Veroni tessellation was set to 1,080 (which is at least three times our sample size) as per Guillot et al. 2005 [58]. We set the spatial coordinate uncertainty (delta.coord value) in GENELAND to 0.0004 decimal degrees based on mean linear home-range of eastern hellbenders [29] to account for errors in individual GPS coordinates and variance in movements of individuals within rivers. The uncorrelated allele frequencies model was selected to accommodate potential uneven, clumped sampling across a relatively large area between rivers and prevent overestimation of *K* [45], especially when the true *K* is unknown [59]. Moreover, GENELAND is known to infer additional substructure at the larger values of *K* under the correlated allele model [49]. GENELAND was run with spatial priors at one million iterations and had thinning at every 100 with post-processing chains consisting of 200–500 burn in for points and population maps, respectively.

## Results

### Range-wide Scale

#### Genetic variation

A total of 1,203 tissue samples were collected from 77 discrete stream reaches (individual streams; average ∼17 samples per reach; range of 2–103 samples per reach) and successfully genotyped across 12 tetranucleotide microsatellite markers. Fourteen thousand two hundred and ninety-nine of 14,436 potential genotypes (99.05%) were obtained across all loci and individuals. Estimates of genetic variation were surprisingly high among stream reaches (individual streams; Table 1). The number of alleles per locus ranged from 14 to 63 (mean of 22.67). There were a relatively small number of private alleles at the regional level, indicating some degree of genetic uniformity at the range-wide scale. Only 3% (30 of 924) of tests for Hardy-Weinberg disequilibrium (across all stream reaches and 12 loci) deviated significantly from expected Hardy-Weinberg disequilibrium when corrected for multiple tests using a standard Bonferroni correction. A few loci (N = 5) exhibited evidence of null alleles but all values (mean = 0.025, range 0–0.12) were relatively low (Table S1). While we detected variation in F_is_ values, the majority were slightly negative, non-significant values indicating some level of heterozygosity excess (high genetic variation) observed across populations. However most F_is_ values observed were close to zero

**Table 1:** Representative collection sites, maximum sample size, genetic diversity estimates: average alleles per locus, (A) number of private alleles, (A_p_) observed heterozygosity, (H_o_), and inbreeding coefficient, (F_is_) for eastern hellbenders across 12 microsatellite loci for range-wide and drainage scale.

General collection site/watershed	N	A	A_p_	H_o_	F_is_
***Rangewide***	*1203*	*6.28*	*-------*	*0.819*	*−0.162*
*Ohio Drainage: IN, WV, OH, PA*	*524*	*15.42*	*14*	*0.794*	*0.072*
*TN drainage NC,GA,TN, VA*	*625*	*21.50*	*87*	*0.829*	*0.076*
Blue River, IN	103	10.67	3	0.791	0.057
Captina Creek, OH	12	7.08	0	0.804	0.011
Northern WV1, WV	15	7.42	1	0.800	0.039
Northern WV2, WV	57	10.0	1	0.773	0.074
PA1, PA	92	10.17	0	0.832	0.02
PA2, PA	38	10.75	0	0.815	0.053
PA3, PA	76	11.59	0	0.811	0.041
PA4, PA	27	8.09	1	0.747	0.058
Western Branch of Susq., PA	9	3.83	1	0.833	−0.012
VA1, VA	77	9.25	0	0.803	−0.006
Gasconade River, MO	14	6.08	0	0.738	0.063
Niangua River, MO	10	5.75	0	0.800	−0.096
Big Piney River, MO	17	6.75	0	0.745	0.014
Little River, TN	49	9.59	0	0.819	0.001
Hiawassee, TN	33	12.42	4	0.872	−0.007
***Drainage (GA)***					
HI1, GA	20	10.10	1	0.858	0.021
HI2, GA	21	9.00	0	0.817	0.019
HI3, GA	20	10.10	1	0.867	0.01
HI4, GA	30	10.10	0	0.853	0.002
HI5, GA	20	9.67	3	0.825	0.033
HI6, GA	20	8.42	0	0.767	0.066
HI7, GA	15	8.42	0	0.843	0.005
HI8, GA	33	7.67	1	0.751	0.045
***Drainage (NC)***					
FB1, NC	31	11.83	1	0.874	−0.02
FB2, NC	26	10.75	2	0.865	−0.015
FB3, NC	13	8.50	0	0.813	0.063
FB4, NC	27	11.17	0	0.854	−0.005
FB5, NC	20	10.33	1	0.817	0.059
FB6, NC	20	10.25	1	0.867	−0.001
FB7, NC	21	10.17	1	0.817	0.032
FB8, NC	15	9.17	0	0.879	−0.015

States listed by abbreviations: IN = Indiana, WV = West Virginia, OH = Ohio, PA = Pennsylvania, TN = Tennessee, VA = Virginia, MO = Missouri, NC = North Carolina, GA = Georgia.

#### Population Genetic Structure

Pairwise F_st_ values between eastern hellbender populations from streams sampled across the species range were generally low but significantly different from panmixia (median = 0.067; range = 0.0009–0.2182; Table S2) The lowest pairwise divergence values among sampling locations were observed for connected stream reaches within sub-basins. Alternatively, eastern hellbenders sampled from streams in Missouri which represent the disjunct portion of the eastern hellbender's range, and those from the West Branch of the Susquehanna River in Pennsylvania which flows into a separate drainage of the Chesapeake Bay, were consistently the most highly differentiated from other eastern hellbender populations throughout the species range (pairwise F_st_ ranges, 0.0817–0.1852; 0.108–0.2118 respectively). Estimates of Jost's D were consistent with F_st_ values.

**Figure 3:**
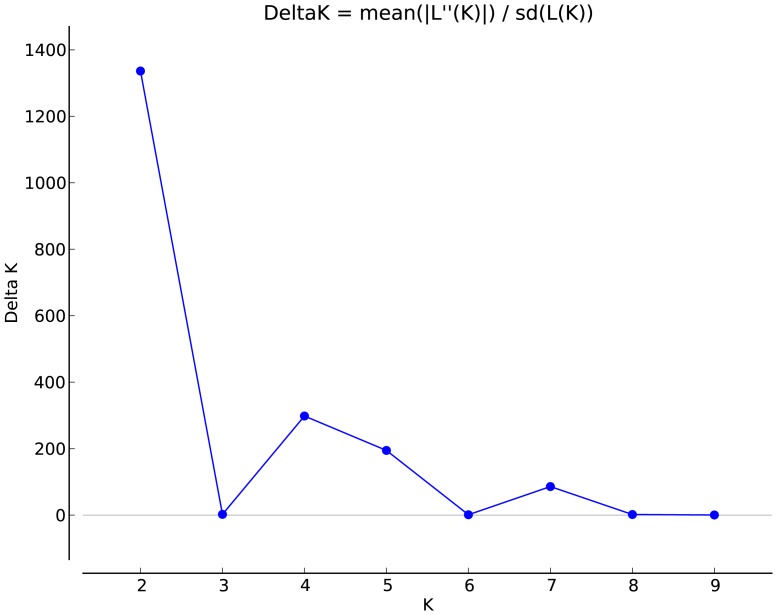
Range-wide plot of mean likelihood values (averaged across runs) for eastern hellbender putative clusters (*K*) obtained from STRUCTURE HARVESTER. Runs include all range-wide individuals and denote high Δ*K* and low variance for mean estimate ln probability of data at *K* = 2.

**Figure 4:**
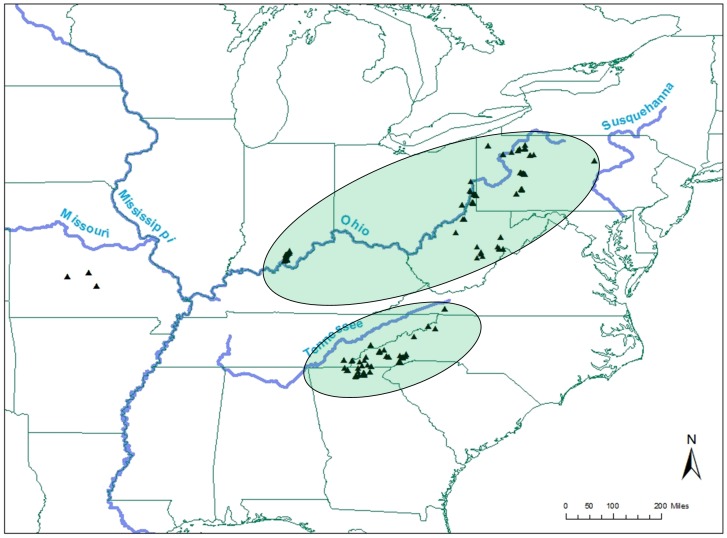
Geographic clusters for range-wide eastern hellbenders according to STRUCTURE (*K* = 2). Circled areas represent distinct genetic clusters of the Ohio River drainage and Tennessee River drainage individuals according to STRUCTURE. Rivers from Missouri are not circled due to the disjunct range (2 rivers clusters as part of Ohio River drainage, while remaining river clustered as part of Tennessee drainage).

The Bayesian clustering analysis implemented in STRUCTURE grouped individuals into two major clusters based on the Δ*K* method of Evanno et al. 2005 ([46]; Figure 3). The pattern of two clusters was consistent regardless of run time. For STRUCTURE *K* = 2, we removed 33 and 21 individuals from Ohio River cluster and the non-Ohio River cluster, respectively, since these individuals were below the 70% threshold. Based on this STRUCTURE analysis, there are two distinct genetic groups at the range-wide scale, a northern group consisting of Ohio River drainage populations, and a southern group consisting of primarily Tennessee River drainage populations (Figure 4). There was a weakly detectable secondary zenith at *K* = 4, indicating some degree of secondary substructure. Further exploration at *K* = 4 found the Ohio drainage grouped into a single cluster, the Tennessee drainage cluster grouped into two clusters, and an additional cluster comprised of individuals from remaining range-wide stream reaches.

### Drainage Scale

**Figure 5:**
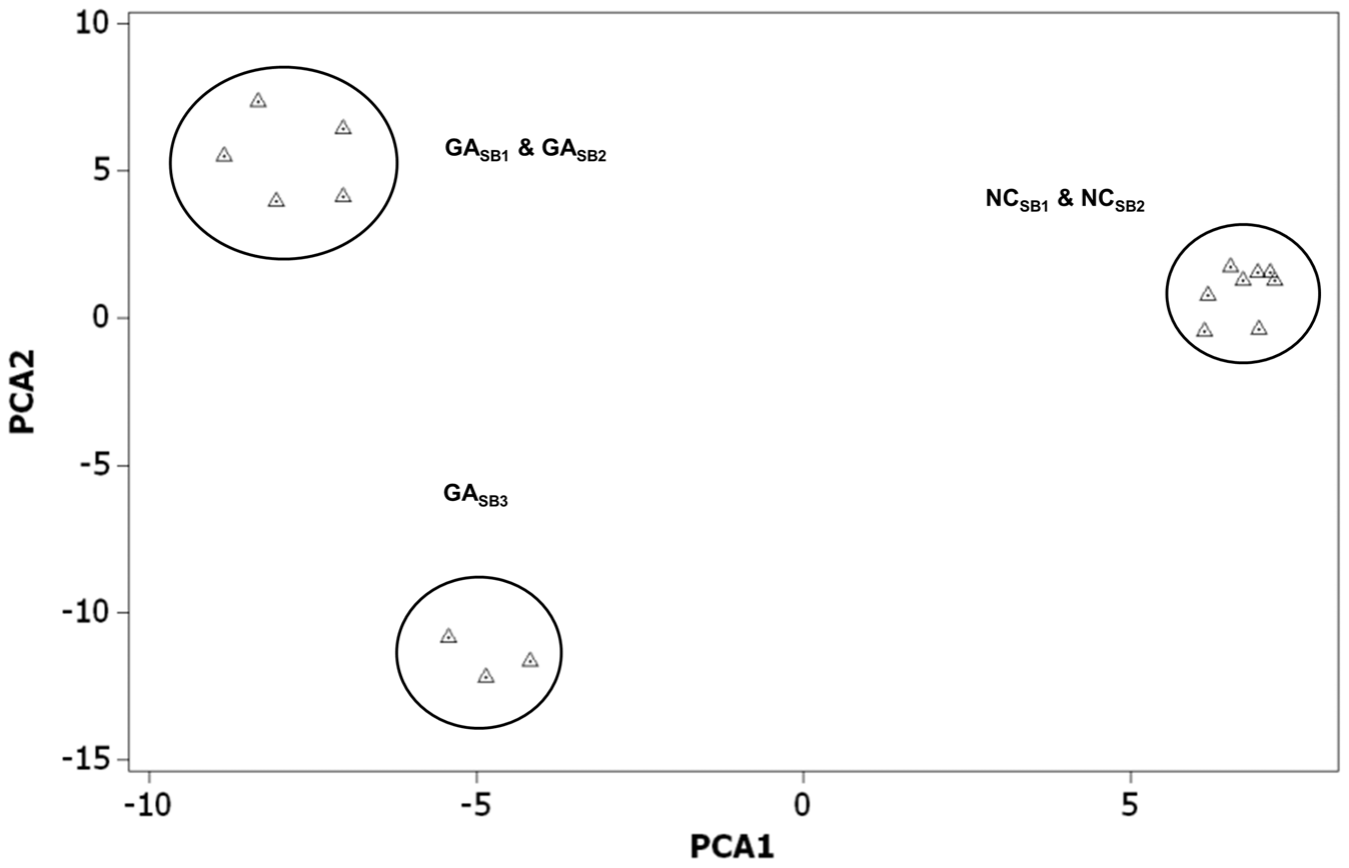
Principle Component Analysis of allele frequencies of eastern hellbenders for 12 microsatellite markers corresponding to streams within sub-basins for landscape scale. Individual stream reaches are represented by triangles: sub-basins are circled. Note grouping of both North Carolina sub-basins together according to basin, while Georgia sub-basins were grouped together in the same basin with the exception of GA_SB3_. The PCA ordination resulted in PC1 and PC2 explaining 20.1% and 15.4% of the variation, respectively.

**Figure 6:**
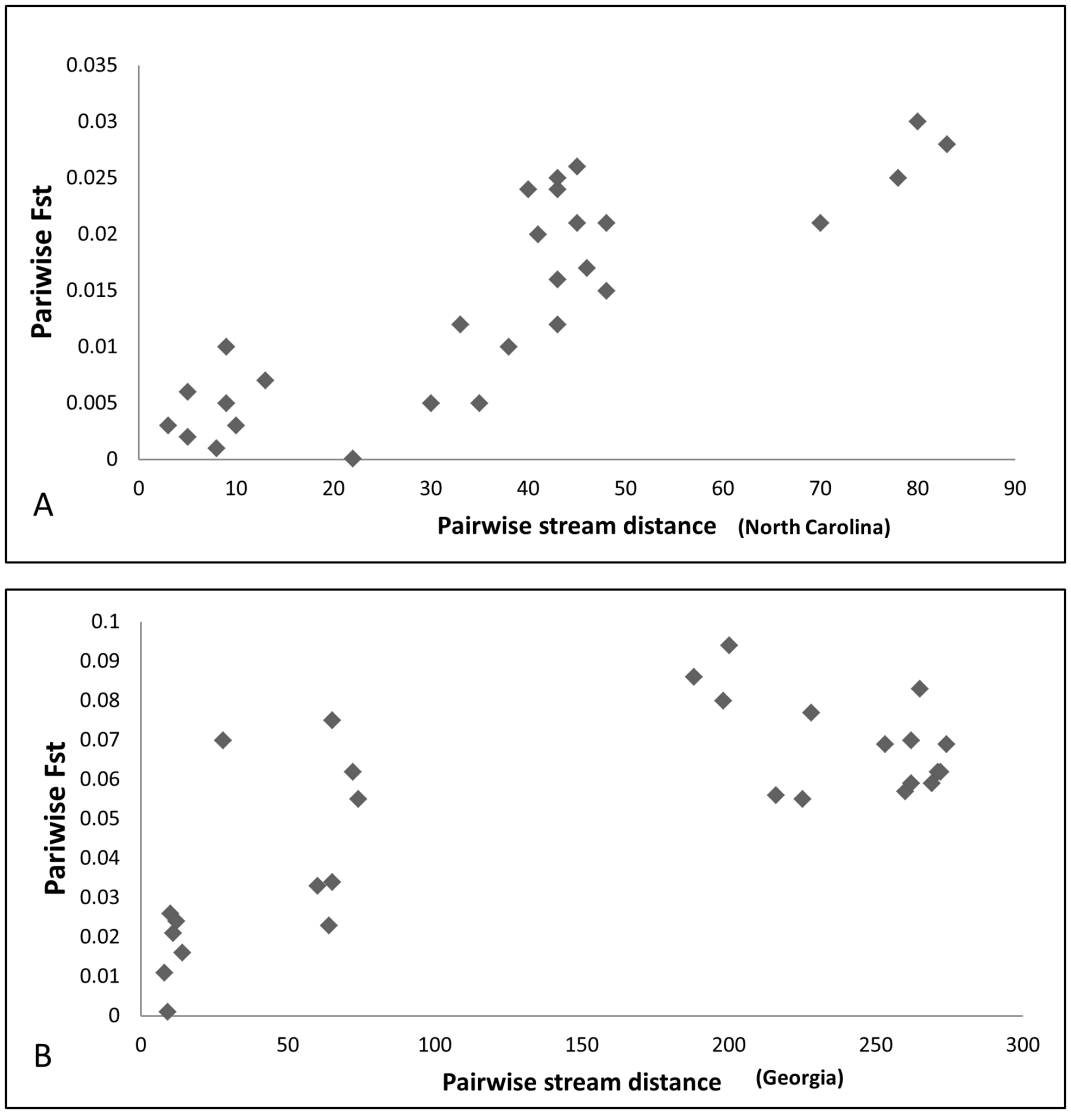
Isolation by stream-distance plot for North Carolina (A) and Georgia (B) streams used in drainage scale study for eastern hellbenders. Linear stream distances between stream reaches in kilometers [(A): R^2^ = 0.715, *P*<0.001; (B): R^2^ = 0.497, *P*<0.001)]. Note lower range of F_st_ values between streams in North Carolina versus Georgia.

The highest degree of genetic variation was partitioned within streams (93.6–98.35%) (Table 2). The level of genetic structuring among sub-basins within basins (1.17–3.71%) and among streams within sub-basins (0.47–2.75%) varied slightly but were overall low for both Georgia and North Carolina AMOVAs. The Global AMOVA resulted in a similar pattern of genetic variance partitioning with 94.93% found within stream reaches and 1.70% found within basins. The PCA ordination for allele frequencies within rivers resulted in PC1 and PC2 explaining 20.1% and 15.4% of the variation, respectively (Figure 5). The first two principle components separated sub-basins into three groups: NC_SB1_ and NC_SB2_, GA_SB1_ and GA_SB2_, and GA_SB3_. This analysis grouped sub-basins into basin groups, with the exception of GA_SB3_, which grouped separate from GA_SB1_ and GA_SB2_. We detected a stronger pattern of isolation by stream distance at the basin scale (Figure 6 (A) & (B); NC: R^2^ = 0.715, *P*<0.001; GA: R^2^ = 0.497, *P*<0.001).

**Table 2:** Drainage scale AMOVA results for hierarchical partitioning of genetic variation on eastern hellbenders for within North Carolina basin, within Georgia basin, and among basins (Global).

Source of Variation	Df	Variance components	Percentage of variation	*F* statistics	*P*	AMOVA comparison
						*North Carolina*
Among sub-basins within basins	1	0.059	1.17	*F*_sb_ = 0.012	*P* = 0.028	
Among stream reaches within sub-basins	6	0.024	0.47	*F*_srsb_ = 0.005	*P*<0.01	
Within stream reaches	338	4.99	98.37	*F*_is_ = 0.016		
						*Georgia*
Among sub-basins within basins	2	0.198	3.71	*F*_sb_ = 0.037	*P*<0.01	
Among stream reaches within sub-basins	5	0.147	2.75	*F*_srsb = _0.029	*P*<0.001	
Within stream reaches	352	5.000	93.54	*F*_is_ = 0.065		
						*Global*
Among Basins	1	0.090	1.70	*F*_b_ = 0.017	*P*<0.01	
Among sub-basins	3	0.177	3.34	*F*_sb_ = 0. 034	*P*<0.001	
Among streams reaches within sub-basins	701	5.049	94.97	*F*_srsb_ = 0.051	*P*<0.001	

Hierarchical AMOVA F statistics are defined as the following; *F*_sb_ = divergence among sub-basins, *F*_srsb_ = divergence among stream reaches, *F*_is_ = inbreeding coefficient within stream reaches, and *F*_b_ = divergence among basins.

**Figure 7:**
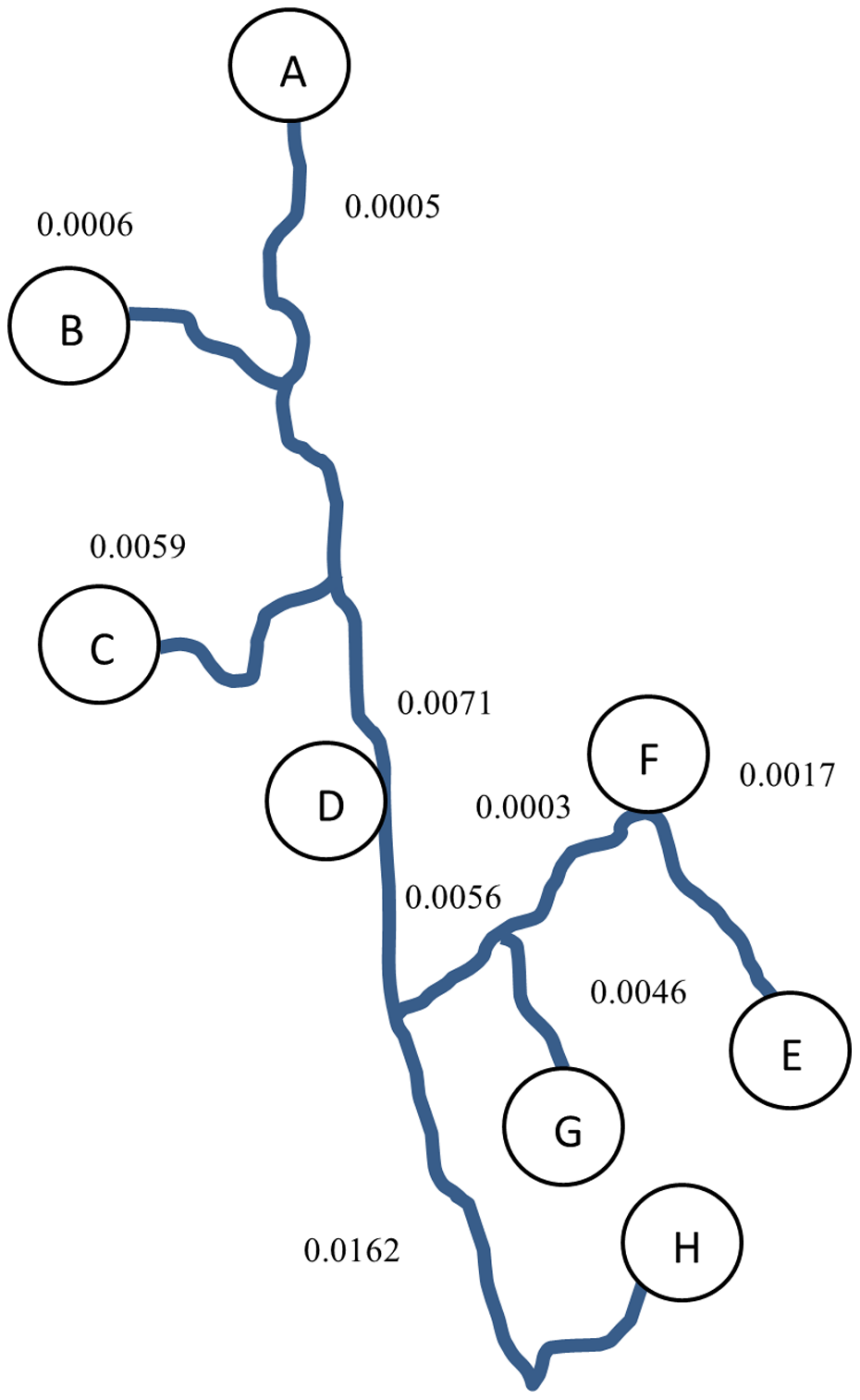
A diagram of STREAMTREE showing the relationship of hierarchical structure of North Carolina sub-basins, NC_SB1_ (E, F, G, H) and NC_SB2_ (A, B, C, D) sampled for eastern hellbenders. Letters correspond to sample locations along stream reaches. The observed R^2^ = 0.852, indicating fit to the STREAMTREE model. Fitted genetic distances are shown for each stream reach according to STREAMTREE (Kalinowski et al. 2008).

There was strong fit of the data to the STREAMTREE model (R^2^ = 0.852), indicating that stream-reach distances and watershed topology correlated well with the corresponding genetic distance matrix (Table 3), which is in agreement with our IBSD analysis within the same North Carolina Basin [56]. The largest genetic distance for a stream section corresponded to a stream separated by a large elevation gradient compared to neighboring streams within the same catchment (Figure 7). Surprisingly, the stream section between our two North Carolina sub-basins showed a relatively low genetic distance (r_6_ = 0.0056).

**Table 3:** Matrix of pairwise F_st_ comparisons used for STREAMTREE analysis for eastern hellbenders.

	A	B	C	D	E	F	G
B	0.006						
C	0.007	0.001					
D	0.001	0.010	0.003				
E	0.016	0.015	0.021	0.005			
F	0.010	0.012	0.024	0.005	0.002		
G	0.020	0.017	0.026	0.012	0.005	0.003	
H	0.025	0.028	0.030	0.021	0.021	0.025	0.024

Letters correspond to sample locations within North Carolina sub-basins NC_SB1_ (A–D) and NC_SB2_ (E–H).

The number of distinct genetic clusters within the drainage scale varied slightly among the clustering programs. STRUCTURE detected 3 distinct genetic clusters: cluster 1 (NC_SB1_, NC_SB2_), cluster 2 (GA_SB1_), and cluster 3 (GA_SB2_ & GA_SB3_). GENELAND results were identical to STRUCTURE for clusters 1 and 2, but partitioned cluster 3 into an additional cluster composed of a single stream reach within GA_SB3_.

## Discussion

### Range-wide

#### Genetic variation

Overall, we found relatively high levels of genetic diversity at the range-wide level. A few rivers had private alleles, but most populations shared allele ranges (the minimum and maximum allele sizes) and exhibited similar levels of allelic richness (Table 1). When taken together, this indicates a high degree of genetic uniformity range-wide. The discrepancy between this high level of genetic diversity and demographic decline is likely a genetic signature of historically larger populations obfuscated by the hellbender's long life span. This apparent time lag and genetic signature of population decline (i.e., decrease in genetic variation, loss of alleles, etc.) has been attributed to adult longevity in a variety of taxa including fish [60], turtles [61], mammals [62], and birds [63]. It follows that species with long life spans and limited dispersal capabilities may retain signatures of genetic variation within localized geographic regions over much longer periods than for species with shorter life spans [64] or high vagility [65]. Indeed, species characterized by low vagility may retain a genetic signal from past events for tens to hundreds of generations [66].

#### Population Genetic Structure

While most of the eastern hellbender stream reaches (populations) sampled within and among drainages were significantly genetically differentiated, overall we found strikingly lower levels of genetic variance partitioning than have been reported in previous studies [31], [32]. This discrepancy in magnitude of genetic variance partitioning may be a result of differences in the genetic variability of the microsatellite markers used among studies, or the high mutation rate loci used in the current study. Overall the magnitude of F_st_ values between populations were congruent with geographic proximity, i.e., geographically proximate rivers and connected rivers within the same watersheds yielded lower F_st_ values. The moderately low F_st_ values between geographically proximate populations were somewhat unexpected as eastern hellbenders are very sedentary and highly philopatric [67], [29]. Adults are documented to move infrequently throughout the year (mean = ∼14 mean movements per year) over relatively short distances (mean = ∼28 m; [29]). Conversely, eastern hellbender populations from the peripheral portions of the range (specifically, Missouri rivers and Western Branch of the Susquehanna in Pennsylvania) were consistently differentiated from the remainder of the North American population. These populations are of particular conservation concern as their isolation indicates a low probability of genetic rescue from adjacent populations.

We identified two major genetic populations at the range-wide scale using Bayesian methods, the Ohio River drainage and Tennessee River drainage. STRUCTURE identified these two distinct clusters (*K* = 2) consistently, regardless of iteration or burn in length, using Δ*K* Evanno et al. 2005 [46], and highest lnP (individual runs and mean at particular run of *K*) with significantly higher assignment probabilities. Latch et al. 2006 [68] found STRUCTURE performed well at low levels of genetic differentiation (F_st_ = 0.03–0.05), which overlaps well with the range of most (i.e. 92% above 0.03) of our values range-wide.

The clustering of Ohio River drainage samples into one population makes biological sense given that the assignments correspond to spatial stream patterns of tributaries that flow directly into the Ohio River. Moreover, the results of our study are similar to the assignment of Ohio River drainage clusters in previous genetic assessments for eastern hellbenders [30], [31]. In contrast, the populations within Tennessee River drainage are distributed across a more physically complex spatial network with greater elevation and gradient changes and higher stream hierarchy (i.e., more tributaries encountered before reaching the mainstem). These results delineating Ohio and Tennessee watersheds represent a tractable scenario for managers, since managing across these two distinct genetic drainages is likely more plausible than managing individual rivers that extend across multiple state boundaries.

### Drainage Scale

For fully aquatic species, dispersal is constrained by riverscape architecture in which terrestrial habitats are barriers to movement [6]. Conversely, lotic species which have biphasic life cycles have additional dispersal opportunities along and across riparian-associated terrestrial habitats. It follows that fully aquatic species should be highly structured and conform to a pattern of greater partitioning of genetic variation at higher hierarchical scales (basin & sub-basins). This scaling-up effect results from divergence of allele frequencies due to genetic drift and other processes which partitions genetic variation among populations [69]. Conversely, lotic species with the ability to move genes across streams through terrestrial dispersal should exhibit low levels of structure and have more of their genetic variation within sub-basins of basins due to greater mixing resulting in more homogenous allele frequencies [70]. This concept of the Stream Hierarchy Model, whereby genetic structure is lowest at the smallest scale (within streams) and greatest at the largest scale (among river sub-basins), has been tested for a relatively small number of species (predominantly fish; [70], aquatic insects [12], and one study on salamanders [11]. This “dendritic ecological framework” [4] in which lotic networks are shaped by ecological processes leading to similarity across hierarchical scales may not apply to species with varying terrestrial affinities.

The greatest partitioning of genetic variation in this study was within streams (∼94–98). The percent of genetic variation partitioned among drainages (∼1.2%–3.7%) and within sub-basins (∼0.5%–2.8%) were both relatively low (although significant). Results were similar for the global AMOVA which partitioned 1.70 percent of the genetic variation among basins. Initial colonization by these obligate aquatic salamanders has been proposed to have occurred toward the end of the Pleistocene as glaciers receded and individuals moved from larger connected rivers to smaller reaches within the stream hierarchical network [30]. It has been shown that fine-scale analysis in recently de-glaciated areas may result in a lack of strong signature due to river connectivity changing over time [71].

The significant genetic structuring detected at higher watershed scales (among basins and within sub-basins of the Tennessee River drainage) should not be discounted as it indicates individual drainages and larger hydrologic features are important in contributing to landscape level genetic sub-structure. Alternatively, a lack of structuring at higher hierarchical levels (i.e. sub-basin) may indicate insufficient time for structure to accrue at this spatial scale given the relatively short time frame following glacial retreat and significant paleo-river reconfiguration in this region [72]. However, the strong correlation between F_st_ and linear stream distance (i.e., IBSD) indicates some effect of localized dispersal among nearby populations. Reported patterns of genetic signal relative to stream hierarchy vary across taxa. For example, there is a clear scaling-up effect of genetic variance partitioning among sub-basins in species with some degree of terrestrial dispersal (Table 4). Indeed, the black-bellied salamander *(Desmognathus quadramaculatus*) conforms well to predictions of higher variance within stream reaches (lower hierarchical levels) possibly due to high levels of overland dispersal [73]. On the other hand, patterns of variance partitioning for some species of fishes are not explained by high dispersal ability, but rather strong natal philopatry within particular rivers or higher historical connectivity [71]. It is surprising that organisms confined to the water column and benthos, including fish, mussels (which rely on their fish host), and eastern hellbenders do not readily conform to predicted patterns of greater variance partitioning among streams [70]. For eastern hellbenders periodic flooding resulting in stream drift of juvenile salamanders downstream [74] may explain this lack of structure among sub-basins. This, along with sub-adult dispersal, could result in higher genetic variation within rivers (high gene flow) than would be expected for highly philopatric lotic species with presumed low dispersal, which would be expected to show greater genetic variation partitioned among streams. The discrepancy between predicted patterns and those observed across taxa indicate the need for further study within lotic systems where gene flow is thought to be constrained by stream hierarchy.

**Table 4:** Comparison of present study to previous studies using hierarchical AMOVA's within basins, sub-basins, and streams with differing dispersal traits.

Taxa	% Variation among sub-basins	% Variation among stream reaches	% variation within stream reaches	Citation	Dispersal biology	Dispersal ability	Philopatry	Total sample size
Brooke char, *Salvelinus fontinalis*	_	−.07	∼100	[71]	Aquatic (anadromous)	Low	High	581
Eastern hellbender, *Cryptobranchus a. alleganiensis*	∼1–4	∼0.5–3	∼94–98	Current study	Aquatic	Low	High	354
Atlantic salmon, *Salmo salar*	2.54	2.02	95.4	[1]	Aquatic (anadromous)	Low	High	2,775
Sea trout, *Salmo trutta*	3.0	5.5	91.5	[78]	Aquatic/Oceanic	Low (anadromous)	High	282
Yellow lampmussel, *Lampsilis cariosa*	4.2	4.4	91.4	[79]	Fully Aquatic (linked to host)	Low	unknown	203
Black-bellied salamander, *Desmognathus quadramaculatus*	------	4.5	90.4	[80]	Aquatic/Terrestrial	Aquatic/terrestrial	unknown	281
Yazoo darter, *Ethostoma raneyi*	7.3	9.2	84.5	[81]	Aquatic	Low	Currently restricted	212
Columbia spotted frog, *Lithobates luteiventris*	17.6	3.8	76.6	[13]	Terrestrial/Aquatic (juvenile)	Moderate	variable	790
Giant salamander, *Dicamptodon aterrimus*	24.3	7.3	68.4	[11]	Aquatic/Terrestrial (riparian adults)	Moderate	unknown	361
Alligator snapping turtle, *Macrochelys temminckii*	-	42.6	57.4	[82]	Aquatic/terrestrial	Moderate	Variable	195
Water bug, *Abedus herberti*	7.52	48.2	44.28	[12]	Aquatic larvae/Terrestrial adult	Moderate	unknown	531

Ranked by percent variation within streams.

Interestingly, the results of the drainage scale analysis using STRUCTURE and GENELAND indicate further fine-scale genetic structuring at hierarchical levels (sub-basins), as both programs grouped North Carolina sub-basins together, but differentiated Georgia sub-basins into proper sub-basins. GENELAND, however further divided one Georgia sub-basin (GA_SB3_) into an additional cluster composed of a single stream reach. This same sub-basin indicated some degree of differentiation (limited gene flow) from other Georgia stream reaches within the same Basin in the PCA analyses. One stream segment in this sub-basin occurs in an area separated by a higher elevational gradient and separated by a greater linear stream distance than other stream reaches within GA_SB1_ and GA_SB2_.

While we observed an isolation by stream distance pattern for both drainage scale basins (Georgia and North Carolina), we observed a more linear trend for North Carolina while Georgia genetic differences appear to increase rapidly at short distances then plateau. This difference in pattern may be due to the shorter stream distances and lower levels of genetic differentiation estimates observed within North Carolina sub-basins than those in Georgia or a lack of intermediate sample locations. Alternatively, this nonlinear pattern may be a result of landscape features other than linear distance (geographic barriers, etc.) influencing genetic differentiation within Georgia sub-basins. Moreover, this nonlinear pattern is supported by the clustering results and may reflect the fine-scale structure of two distinct Georgia sub-basins. When taken together, these results reveal the presence of fine-scale structure at the drainage scale characterized by detectable level of IBSD.

Future management of the eastern hellbender across these two major drainages (Ohio and Tennessee Rivers) presents many challenges. Many populations have declined to the point where the only remaining viable management tools are captive propagation and translocations, both of which require understanding of the genetic and biological attributes of source and target populations if they are to be successful. Translocation programs may suffer from low success rates if they fail to incorporate underlying levels of genetic structure [75] or rely on stock populations characterized by low genetic diversity [76], [77]. The results of our study are encouraging, however, as many rivers retain high genetic diversity. In several cases we found as much genetic structure within basins as we did across drainages (Table S2). For eastern hellbenders, future translocations within individual watersheds should focus on sub-basins within the lower range of genetic differentiation (Table S2). If source populations are unavailable within sub-basins, as may be the case for several isolated populations within the Ohio River drainage, care should be taken to identify source stocks from other tributaries of the mainstem Ohio River. Based on the results of our range-wide study, we recommend management of distinct Ohio River and Tennessee River drainage populations to maintain genetic integrity and evolutionary trajectory. Based on the results of the landscape drainage scale (hierarchical) study, maintenance of individual stream genetic diversity within sub-basins should also be considered. Future genetic studies should focus on the effects of potential fragmenting landscape features (e.g., dams, degraded habitat as streams barriers, etc.) on fine-scale genetic structure.

## Supporting Information

Table S1 **Locus-specific information for range-wide study.** Null allele presence (statistically significant in *) and frequencies for all eastern hellbender populations. The number of alleles observed at each locus is reported along with loci-specific F_is_.
(DOCX)

Table S2 **F_st_ and Jost's D matrix of Representative eastern hellbender populations with minimal sample size of 8.** Pairwise F_st_ values are below the diagonal, Jost's D values are above. F_st_ values that are not significant are in bold. Abbreviations as follows; IN (Blue River, Indiana), HI1 (HI1, Georgia), HI2 (HI2, GA), HI3 (HI3, Georgia), HI4(HI4, Georgia), HI5 (HI5, Tennessee), HI6 (HI6, Georgia), HI7 (HI7, Georgia), HI8 (HI8, Georgia), HI9 (HI9, Georgia), HI10 (HI10, Georgia), LT (Little River, Tennessee), Elk (Elk Creek, TN), Cap (Captina Creek, Ohio), MO1 (Gasconade River, Missouri), MO2 (Niangua River, Missouri), MO3 (Big Piney River, Missouri), VA1 (VA1, Virginia), WV1(Northern WV1, West Virginia), WV2 (Northern WV2, West Virginia), PA1 (PA1, Pennsylvania), PA2 (PA2, Pennsylvania), PA3 (PA3, Pennsylvania), PA4 (PA4, Pennsylvania), PA5 (PA5, Pennsylvania), PA6 (PA6, Pennsylvania), PA7 (PA7, Pennsylvania), PA8 (PA8, Pennsylvania), FB1 (FB1, North Carolina), FB2 (FB2, North Carolina), FB3 (FB3, North Carolina), FB4 (FB4, North Carolina), Deep (Deep, North Carolina), Tuck (Tuck, North Carolina), FB5 (FB5, North Carolina), FB6 (FB6, North Carolina), New1 (New1, North Carolina), New2 (New2, North Carolina), New3 (New3, North Carolina), New4 (New4, North Carolina), New5 (New5, North Carolina), FB7 (FB7, North Carolina), FB8 (FB8, North Carolina).
(DOCX)
