# Supplementary material for: Population Genetics of the Eastern Hellbender (Cryptobranchus alleganiensis alleganiensis) across Multiple Spatial Scales
Source: PLoS One. 2013 Oct 18;8(10):e74180. doi: 10.1371/journal.pone.0074180 (PMC3800131; doi:10.1371/journal.pone.0074180)
Supplement: Table S1 — Locus-specific information for range-wide study. Null allele presence (statistically significant in *) and frequencies for all eastern hellbender populations. The number of alleles observed at each locus is reported along with loci-specific Fis. (DOCX) [file pone.0074180.s001.docx]

**Table S1**. **Locus-specific information for range-wide study**.

| Locus | Null allele frequency | Allelic richness | F_is_ |
| --- | --- | --- | --- |
| Call171 | 0.065* | 21 | -0.195 |
| Call127 | 0.049* | 14 | -0.149 |
| Call351 | 0.049* | 18 | -0.127 |
| Call204 | 0.026 | 21 | -0.122 |
| Call205 | 0.071* | 19 | -0.191 |
| Call232 | -0.028 | 63 | -0.151 |
| Call347 | -0.010 | 17 | -0.127 |
| Call282 | -0.002 | 16 | -0.192 |
| Call341 | -0.007 | 18 | -0.207 |
| Call261 | 0.102* | 16 | -0.175 |
| Call26 | -0.025 | 23 | -0.131 |
| Call266 | 0.012 | 26 | -0.172 |
