# Supplementary material for: Population Genetics of the Eastern Hellbender (Cryptobranchus alleganiensis alleganiensis) across Multiple Spatial Scales
Source: PLoS One. 2013 Oct 18;8(10):e74180. doi: 10.1371/journal.pone.0074180 (PMC3800131; doi:10.1371/journal.pone.0074180)
Supplement: Table S2 — Fst and Jost's D matrix of Representative eastern hellbender populations with minimal sample size of 8. Pairwise Fst values are below the diagonal, Jost's D values are above. Fst values that are not significant are in bold. Abbreviations as follows; IN (Blue River, Indiana), HI1 (HI1, Georgia), HI2 (HI2, GA), HI3 (HI3, Georgia), HI4(HI4, Georgia), HI5 (HI5, Tennessee), HI6 (HI6, Georgia), HI7 (HI7, Georgia), HI8 (HI8, Georgia), HI9 (HI9, Georgia), HI10 (HI10, Georgia), LT (Little River, Tennessee), Elk (Elk Creek, TN), Cap (Captina Creek, Ohio), MO1 (Gasconade River, Missouri), MO2 (Niangua River, Missouri), MO3 (Big Piney River, Missouri), VA1 (VA1, Virginia), WV1(Northern WV1, West Virginia), WV2 (Northern WV2, West Virginia), PA1 (PA1, Pennsylvania), PA2 (PA2, Pennsylvania), PA3 (PA3, Pennsylvania), PA4 (PA4, Pennsylvania), PA5 (PA5, Pennsylvania), PA6 (PA6, Pennsylvania), PA7 (PA7, Pennsylvania), PA8 (PA8, Pennsylvania), FB1 (FB1, North Carolina), FB2 (FB2, North Carolina), FB3 (FB3, North Carolina), FB4 (FB4, North Carolina), Deep (Deep, North Carolina), Tuck (Tuck, North Carolina), FB5 (FB5, North Carolina), FB6 (FB6, North Carolina), New1 (New1, North Carolina), New2 (New2, North Carolina), New3 (New3, North Carolina), New4 (New4, North Carolina), New5 (New5, North Carolina), FB7 (FB7, North Carolina), FB8 (FB8, North Carolina). (DOCX) [file pone.0074180.s002.docx]

**Table S2.** **F_st_ and Jost’s D matrix of Representative eastern hellbender populations with minimal sample size of 8.** Pairwise F_st_ values are below the diagonal, Jost’s D values are above. F_st_ values that are not significant are in bold.

101

| **Pop** | **IN** | **HI1** | **HI2** | **HI3** | **HI4** | **HI5** | **HI6** | **HI7** | **HI8** | **HI9** | **HI10** | **LT** | **Elk** | **Cap** |
| --- | --- | --- | --- | --- | --- | --- | --- | --- | --- | --- | --- | --- | --- | --- |
| **IN** |  | 0.276 | 0.254 | 0.258 | 0.195 | 0.199 | 0.262 | 0.369 | 0.303 | 0.236 | 0.385 | 0.265 | 0.167 | 0.177 |
| **HI1** | 0.066 |  | 0.195 | 0.176 | 0.047 | 0.038 | 0.241 | 0.051 | 0.011 | 0.26 | 0.348 | 0.227 | 0.189 | 0.231 |
| **HI2** | 0.064 | 0.057 |  | 0.026 | 0.201 | 0.182 | 0.075 | 0.233 | 0.252 | 0.111 | 0.236 | 0.227 | 0.186 | 0.267 |
| **HI3** | 0.065 | 0.059 | 0.011 |  | 0.177 | 0.142 | 0.046 | 0.268 | 0.25 | 0.068 | 0.225 | 0.327 | 0.228 | 0.25 |
| **HI4** | 0.053 | 0.016 | 0.05 | 0.052 |  | 0.007 | 0.157 | 0.08 | 0.041 | 0.218 | 0.266 | 0.195 | 0.211 | 0.262 |
| **HI5** | 0.049 | 0.018 | 0.046 | 0.045 | **0.005** |  | 0.203 | 0.104 | 0.026 | 0.223 | 0.25 | 0.152 | 0.209 | 0.187 |
| **HI6** | 0.075 | 0.069 | 0.026 | **0.021** | 0.056 | 0.06 |  | 0.304 | 0.255 | 0.097 | 0.209 | 0.242 | 0.171 | 0.265 |
| **HI7** | 0.08 | 0.024 | 0.062 | 0.069 | 0.025 | 0.026 | 0.083 |  | 0.036 | 0.355 | 0.359 | 0.266 | 0.263 | 0.282 |
| **HI8** | **0.066** | 0.01 | 0.059 | 0.062 | **0.014** | **0.007** | 0.07 | **0.016** |  | 0.31 | 0.302 | 0.22 | 0.243 | 0.252 |
| **HI9** | 0.063 | 0.056 | 0.034 | 0.023 | 0.047 | 0.044 | 0.033 | 0.077 | 0.055 |  | 0.297 | 0.236 | 0.246 | 0.287 |
| **HI10** | 0.091 | 0.086 | 0.062 | 0.055 | 0.074 | 0.072 | 0.075 | 0.094 | 0.08 | 0.069 |  | 0.305 | 0.285 | 0.401 |
| **LT** | 0.071 | 0.067 | 0.07 | 0.089 | 0.058 | 0.052 | 0.085 | 0.076 | 0.06 | 0.073 | 0.101 |  | 0.25 | 0.296 |
| **Elk** | 0.049 | 0.05 | 0.067 | 0.074 | 0.05 | 0.046 | 0.072 | 0.067 | 0.056 | 0.077 | 0.09 | 0.084 |  | 0.201 |
| **Cap** | 0.052 | 0.059 | 0.07 | 0.068 | 0.063 | 0.053 | 0.081 | 0.079 | 0.061 | 0.069 | 0.111 | 0.085 | 0.071 |  |
| **MO1** | 0.057 | 0.086 | 0.089 | 0.077 | 0.087 | 0.078 | 0.086 | 0.11 | 0.088 | 0.086 | 0.115 | 0.117 | 0.105 | 0.098 |
| **MO2** | 0.112 | 0.112 | 0.082 | 0.068 | 0.113 | 0.108 | 0.077 | 0.139 | 0.114 | 0.089 | 0.131 | 0.136 | 0.135 | **0.131** |
| **MO3** | 0.078 | 0.095 | 0.09 | 0.075 | 0.092 | 0.088 | 0.092 | 0.109 | 0.096 | 0.089 | 0.111 | 0.135 | 0.118 | 0.111 |
| **VA1** | 0.075 | 0.079 | 0.103 | 0.102 | 0.067 | 0.065 | 0.107 | 0.078 | 0.078 | 0.109 | 0.114 | 0.083 | 0.065 | 0.111 |
| **WV1** | 0.033 | 0.06 | 0.065 | 0.065 | 0.056 | 0.052 | 0.08 | 0.078 | 0.063 | 0.07 | 0.102 | 0.069 | 0.067 | 0.046 |
| **WV2** | 0.024 | 0.062 | 0.058 | 0.056 | 0.052 | 0.044 | 0.06 | 0.071 | 0.054 | 0.059 | 0.082 | 0.063 | 0.074 | 0.046 |
| **PA1** | 0.057 | 0.096 | 0.083 | 0.081 | 0.077 | 0.063 | 0.105 | 0.098 | 0.078 | 0.084 | 0.122 | 0.078 | 0.083 | 0.052 |
| **PA2** | 0.029 | 0.062 | 0.056 | 0.064 | 0.044 | 0.041 | 0.072 | 0.069 | 0.055 | 0.069 | 0.091 | 0.067 | 0.061 | 0.047 |
| **PA3** | 0.027 | 0.059 | 0.063 | 0.064 | 0.043 | 0.04 | 0.073 | 0.072 | 0.051 | 0.069 | 0.091 | 0.057 | 0.057 | 0.034 |
| **PA4** | 0.027 | 0.038 | 0.054 | 0.056 | 0.034 | 0.029 | 0.065 | 0.053 | 0.036 | 0.055 | 0.089 | 0.047 | 0.046 | 0.03 |
| **PA5** | 0.056 | 0.102 | 0.098 | 0.103 | 0.08 | 0.076 | 0.125 | 0.111 | 0.094 | 0.113 | 0.145 | 0.096 | 0.084 | 0.071 |
| **PA6** | **0.039** | 0.074 | 0.078 | 0.076 | 0.071 | 0.057 | 0.087 | 0.092 | 0.074 | 0.09 | 0.119 | 0.08 | 0.077 | **0.076** |
| **PA7** | 0.033 | 0.054 | 0.059 | 0.061 | 0.043 | 0.036 | 0.066 | 0.063 | 0.042 | 0.056 | 0.091 | 0.068 | 0.046 | **0.035** |
| **PA8** | 0.119 | 0.157 | 0.152 | 0.145 | 0.132 | 0.134 | 0.127 | 0.179 | 0.149 | 0.147 | 0.214 | 0.159 | 0.176 | **0.185** |
| **FB1** | 0.047 | 0.043 | 0.063 | 0.062 | 0.037 | 0.028 | 0.071 | 0.059 | 0.031 | 0.055 | 0.091 | 0.063 | 0.063 | 0.065 |
| **FB2** | 0.045 | 0.048 | 0.058 | 0.055 | 0.041 | 0.033 | 0.07 | 0.063 | 0.041 | 0.054 | 0.084 | 0.069 | 0.067 | 0.063 |
| **FB3** | 0.054 | 0.046 | 0.062 | 0.065 | 0.038 | 0.034 | 0.082 | 0.058 | 0.039 | 0.059 | 0.089 | 0.066 | 0.069 | 0.089 |
| **FB4** | 0.045 | 0.054 | 0.061 | 0.061 | 0.041 | 0.036 | 0.079 | 0.068 | 0.045 | 0.067 | 0.1 | 0.069 | 0.058 | 0.073 |
| **Deep** | 0.056 | 0.039 | 0.06 | 0.069 | 0.032 | 0.023 | 0.091 | 0.055 | 0.035 | 0.064 | 0.09 | 0.057 | 0.063 | 0.07 |
| **Tuck** | 0.051 | 0.044 | 0.058 | 0.063 | 0.038 | 0.031 | 0.079 | 0.057 | 0.037 | 0.066 | 0.093 | 0.052 | 0.051 | 0.081 |
| **FB5** | 0.041 | 0.036 | 0.053 | 0.049 | 0.036 | 0.026 | 0.062 | 0.049 | 0.028 | 0.053 | 0.088 | 0.06 | 0.052 | 0.054 |
| **FB6** | 0.052 | 0.046 | 0.059 | 0.061 | 0.043 | 0.04 | 0.067 | 0.065 | 0.04 | 0.059 | 0.082 | 0.073 | 0.063 | 0.067 |

| **New1** | 0.089 | 0.107 | 0.112 | 0.112 | 0.098 | 0.091 | 0.11 | 0.118 | 0.108 | 0.116 | 0.149 | 0.116 | 0.06 | 0.082 |
| --- | --- | --- | --- | --- | --- | --- | --- | --- | --- | --- | --- | --- | --- | --- |
| **New2** | 0.064 | 0.039 | 0.066 | 0.077 | **0.035** | 0.046 | 0.074 | 0.071 | **0.039** | 0.055 | 0.097 | 0.053 | 0.085 | **0.077** |
| **New3** | 0.075 | 0.076 | 0.067 | 0.053 | 0.059 | 0.058 | 0.069 | 0.08 | 0.071 | 0.059 | 0.091 | 0.076 | 0.092 | **0.099** |
| **New4** | 0.033 | 0.037 | 0.057 | 0.057 | 0.032 | 0.026 | 0.048 | 0.055 | 0.034 | 0.055 | 0.093 | 0.072 | 0.042 | **0.071** |
| **New5** | 0.09 | 0.079 | 0.077 | 0.064 | 0.055 | 0.065 | 0.087 | 0.075 | 0.084 | 0.07 | 0.117 | 0.102 | 0.105 | **0.12** |
| **FB7** | 0.043 | 0.031 | 0.055 | 0.057 | 0.031 | 0.027 | 0.06 | 0.056 | 0.029 | 0.052 | 0.089 | 0.059 | 0.057 | 0.059 |
| **FB8** | 0.037 | 0.034 | 0.053 | 0.048 | 0.035 | 0.024 | 0.056 | 0.052 | 0.027 | 0.056 | 0.077 | 0.058 | 0.053 | 0.057 |

103

| **MO1** | **MO2** | **MO3** | **VA** | **WV1** | **WV2** | **PA1** | **PA2** | **PA3** | **PA4** | **PA5** | **PA6** | **PA7** | **PA8** | **FB1** |
| --- | --- | --- | --- | --- | --- | --- | --- | --- | --- | --- | --- | --- | --- | --- |
| 0.133 | 0.197 | 0.163 | 0.263 | 0.158 | 0.072 | 0.167 | 0.128 | 0.108 | 0.125 | 0.148 | 0.151 | 0.099 | 0.377 | 0.155 |
| 0.401 | 0.147 | 0.339 | 0.241 | 0.293 | 0.197 | 0.389 | 0.304 | 0.281 | 0.157 | 0.453 | 0.355 | 0.173 | 0.571 | 0.164 |
| 0.319 | 0.19 | 0.278 | 0.332 | 0.268 | 0.22 | 0.259 | 0.205 | 0.222 | 0.192 | 0.328 | 0.297 | 0.186 | 0.523 | 0.242 |
| 0.271 | 0.119 | 0.175 | 0.364 | 0.235 | 0.196 | 0.251 | 0.228 | 0.236 | 0.219 | 0.358 | 0.297 | 0.234 | 0.47 | 0.235 |
| 0.361 | 0.197 | 0.294 | 0.165 | 0.287 | 0.181 | 0.323 | 0.201 | 0.176 | 0.154 | 0.281 | 0.312 | 0.144 | 0.407 | 0.109 |
| 0.312 | 0.174 | 0.264 | 0.18 | 0.279 | 0.172 | 0.271 | 0.171 | 0.174 | 0.136 | 0.276 | 0.252 | 0.127 | 0.458 | 0.088 |
| 0.235 | 0.137 | 0.231 | 0.283 | 0.269 | 0.174 | 0.302 | 0.237 | 0.212 | 0.183 | 0.389 | 0.269 | 0.138 | 0.328 | 0.212 |
| 0.496 | 0.325 | 0.367 | 0.226 | 0.371 | 0.282 | 0.362 | 0.322 | 0.329 | 0.217 | 0.391 | 0.419 | 0.203 | 0.604 | 0.238 |
| 0.351 | 0.207 | 0.285 | 0.261 | 0.362 | 0.224 | 0.32 | 0.249 | 0.242 | 0.191 | 0.37 | 0.393 | 0.187 | 0.489 | 0.11 |
| 0.313 | 0.224 | 0.251 | 0.389 | 0.286 | 0.234 | 0.291 | 0.279 | 0.278 | 0.223 | 0.414 | 0.343 | 0.186 | 0.379 | 0.239 |
| 0.371 | 0.294 | 0.323 | 0.351 | 0.394 | 0.316 | 0.429 | 0.364 | 0.346 | 0.366 | 0.483 | 0.409 | 0.328 | 0.686 | 0.341 |
| 0.448 | 0.256 | 0.443 | 0.283 | 0.242 | 0.196 | 0.229 | 0.257 | 0.192 | 0.159 | 0.307 | 0.286 | 0.184 | 0.475 | 0.167 |
| 0.285 | 0.265 | 0.288 | 0.143 | 0.248 | 0.257 | 0.255 | 0.231 | 0.231 | 0.178 | 0.235 | 0.262 | 0.141 | 0.526 | 0.228 |
| 0.218 | 0.275 | 0.251 | 0.359 | 0.146 | 0.146 | 0.13 | 0.197 | 0.093 | 0.114 | 0.174 | 0.272 | 0.093 | 0.513 | 0.191 |
|  | 0.241 | 0.016 | 0.365 | 0.314 | 0.14 | 0.263 | 0.186 | 0.218 | 0.235 | 0.277 | 0.317 | 0.258 | 0.437 | 0.256 |
| 0.14 |  | 0.148 | 0.288 | 0.266 | 0.191 | 0.321 | 0.211 | 0.156 | 0.126 | 0.452 | 0.173 | 0.098 | 0.334 | 0.148 |
| **0.02** | 0.132 |  | 0.298 | 0.344 | 0.195 | 0.274 | 0.244 | 0.224 | 0.223 | 0.32 | 0.382 | 0.245 | 0.499 | 0.156 |
| 0.125 | 0.148 | 0.125 |  | 0.259 | 0.293 | 0.389 | 0.267 | 0.204 | 0.156 | 0.264 | 0.196 | 0.143 | 0.413 | 0.238 |
| 0.084 | 0.116 | 0.106 | 0.079 |  | 0.064 | 0.146 | 0.107 | 0.076 | 0.064 | 0.186 | 0.091 | 0.05 | 0.418 | 0.2716 |
| 0.062 | 0.11 | 0.075 | 0.088 | 0.029 |  | 0.072 | 0.09 | 0.053 | 0.0625 | 0.175 | 0.083 | 0.022 | 0.309 | 0.179 |
| 0.101 | 0.158 | 0.117 | 0.111 | 0.057 | 0.034 |  | 0.178 | 0.109 | 0.108 | 0.029 | 0.225 | 0.13 | 0.413 | 0.276 |
| 0.068 | 0.116 | 0.088 | 0.078 | 0.033 | 0.033 | 0.052 |  | 0.076 | 0.0819 | 0.179 | 0.129 | 0.081 | 0.417 | 0.203 |
| 0.078 | 0.107 | 0.09 | 0.065 | 0.023 | 0.023 | 0.036 | 0.018 |  | 0.013 | 0.101 | 0.035 | 0.005 | 0.366 | 0.186 |
| 0.073 | 0.095 | 0.086 | 0.054 | 0.02 | 0.025 | 0.036 | 0.02 | **0.006** |  | 0.16 | 0.041 | 0.001 | 0.364 | 0.142 |
| 0.118 | 0.185 | 0.14 | 0.11 | 0.054 | 0.065 | **0.025** | 0.055 | 0.04 | 0.049 |  | 0.195 | 0.185 | 0.411 | 0.333 |
| 0.107 | 0.122 | 0.137 | 0.087 | **0.031** | **0.035** | 0.073 | 0.038 | **0.016** | **0.024** | 0.077 |  | 0.013 | 0.276 | 0.258 |
| 0.084 | 0.099 | 0.093 | 0.073 | 0.032 | **0.026** | 0.05 | 0.024 | **0.006** | **0.002** | **0.054** | **0.016** |  | 0.339 | 0.175 |
| 0.185 | 0.165 | 0.206 | 0.16 | 0.133 | 0.108 | 0.166 | 0.13 | 0.119 | 0.118 | 0.193 | 0.139 | 0.131 |  | 0.387 |
| 0.078 | 0.108 | 0.081 | 0.085 | 0.064 | 0.053 | 0.073 | 0.052 | 0.052 | 0.039 | 0.092 | 0.071 | 0.052 | 0.135 |  |
| 0.078 | 0.114 | 0.086 | 0.092 | 0.064 | 0.05 | 0.08 | 0.057 | 0.056 | 0.047 | 0.098 | 0.075 | 0.058 | 0.133 | **0.002** |
| 0.095 | 0.116 | 0.09 | 0.086 | 0.07 | 0.058 | 0.089 | 0.063 | 0.059 | 0.048 | 0.099 | 0.071 | 0.065 | 0.153 | **0.005** |
| 0.083 | 0.114 | 0.094 | 0.07 | 0.056 | 0.054 | 0.074 | 0.059 | 0.049 | 0.037 | 0.081 | 0.061 | 0.046 | 0.129 | 0.022 |
| 0.102 | 0.122 | 0.106 | 0.068 | 0.062 | 0.059 | 0.079 | 0.053 | 0.048 | 0.03 | 0.075 | 0.06 | 0.039 | 0.174 | 0.05 |
| 0.101 | 0.117 | 0.117 | 0.052 | 0.049 | 0.053 | 0.074 | 0.055 | 0.048 | 0.031 | 0.081 | 0.051 | 0.038 | 0.149 | 0.042 |
| 0.063 | 0.112 | 0.077 | 0.082 | 0.043 | 0.041 | 0.062 | 0.047 | 0.043 | 0.028 | 0.078 | 0.055 | 0.038 | 0.14 | **0.01** |
| 0.073 | 0.121 | 0.088 | 0.096 | 0.057 | 0.055 | 0.075 | 0.056 | 0.051 | 0.042 | 0.097 | 0.072 | 0.043 | 0.141 | 0.024 |

| 0.148 | 0.172 | 0.159 | 0.129 | 0.094 | 0.106 | 0.107 | 0.097 | 0.089 | 0.085 | 0.127 | 0.144 | 0.09 | 0.212 | 0.107 |
| --- | --- | --- | --- | --- | --- | --- | --- | --- | --- | --- | --- | --- | --- | --- |
| 0.104 | 0.128 | 0.113 | 0.103 | 0.064 | 0.052 | 0.08 | 0.063 | 0.061 | 0.043 | 0.097 | **0.085** | 0.057 | **0.158** | 0.047 |
| 0.105 | 0.147 | 0.128 | 0.111 | 0.083 | 0.071 | 0.094 | 0.079 | 0.08 | 0.068 | **0.125** | **0.096** | 0.086 | 0.201 | 0.076 |
| 0.081 | 0.094 | 0.095 | 0.073 | 0.042 | 0.039 | 0.086 | 0.044 | 0.037 | 0.027 | 0.107 | **0.033** | **0.038** | 0.109 | 0.038 |
| 0.116 | 0.178 | 0.116 | 0.114 | 0.099 | 0.089 | 0.121 | 0.094 | 0.095 | 0.084 | 0.153 | 0.116 | 0.108 | 0.204 | 0.085 |
| 0.075 | 0.109 | 0.09 | 0.086 | 0.047 | 0.044 | 0.071 | 0.046 | 0.039 | 0.029 | 0.088 | **0.059** | 0.038 | 0.128 | **0.012** |
| 0.068 | 0.11 | 0.077 | 0.078 | 0.045 | 0.038 | 0.065 | 0.044 | 0.038 | 0.029 | 0.085 | 0.059 | 0.038 | 0.137 | **0.005** |

| **FB2** | **FB3** | **FB4** | **Deep** | **Tuck** | **FB5** | **FB6** | **New1** | **New2** | **New3** | **New4** | **New5** | **FB7** | **FB8** |
| --- | --- | --- | --- | --- | --- | --- | --- | --- | --- | --- | --- | --- | --- |
| 0.156 | 0.195 | 0.161 | 0.2 | 0.263 | 0.118 | 0.154 | 0.256 | 0.235 | 0.269 | 0.139 | 0.362 | 0.143 | 0.109 |
| 0.145 | 0.143 | 0.221 | 0.12 | 0.19 | 0.133 | 0.165 | 0.231 | 0.092 | 0.301 | 0.164 | 0.28 | 0.113 | 0.114 |
| 0.225 | 0.259 | 0.233 | 0.263 | 0.266 | 0.201 | 0.213 | 0.269 | 0.249 | 0.179 | 0.214 | 0.308 | 0.223 | 0.166 |
| 0.161 | 0.234 | 0.215 | 0.326 | 0.262 | 0.132 | 0.188 | 0.268 | 0.245 | 0.161 | 0.302 | 0.221 | 0.171 | 0.126 |
| 0.116 | 0.095 | 0.113 | 0.123 | 0.138 | 0.122 | 0.148 | 0.228 | 0.073 | 0.217 | 0.123 | 0.179 | 0.099 | 0.115 |
| 0.103 | 0.099 | 0.135 | 0.098 | 0.116 | 0.081 | 0.131 | 0.207 | 0.108 | 0.206 | 0.093 | 0.225 | 0.083 | 0.063 |
| 0.208 | 0.302 | 0.268 | 0.364 | 0.297 | 0.159 | 0.155 | 0.225 | 0.204 | 0.113 | 0.136 | 0.296 | 0.135 | 0.117 |
| 0.245 | 0.213 | 0.282 | 0.234 | 0.235 | 0.203 | 0.259 | 0.254 | 0.245 | 0.309 | 0.225 | 0.228 | 0.235 | 0.191 |
| 0.142 | 0.132 | 0.189 | 0.159 | 0.158 | 0.102 | 0.122 | 0.274 | 0.141 | 0.251 | 0.176 | 0.316 | 0.103 | 0.075 |
| 0.189 | 0.235 | 0.316 | 0.303 | 0.351 | 0.219 | 0.21 | 0.374 | 0.141 | 0.212 | 0.254 | 0.218 | 0.223 | 0.242 |
| 0.299 | 0.289 | 0.391 | 0.341 | 0.336 | 0.331 | 0.261 | 0.497 | 0.319 | 0.275 | 0.341 | 0.359 | 0.307 | 0.235 |
| 0.199 | 0.174 | 0.221 | 0.172 | 0.181 | 0.186 | 0.208 | 0.294 | 0.136 | 0.185 | 0.299 | 0.352 | 0.187 | 0.155 |
| 0.259 | 0.276 | 0.199 | 0.224 | 0.183 | 0.168 | 0.189 | 0.132 | 0.279 | 0.248 | 0.176 | 0.319 | 0.179 | 0.148 |
| 0.212 | 0.388 | 0.287 | 0.272 | 0.382 | 0.156 | 0.225 | 0.234 | 0.289 | 0.303 | 0.239 | 0.47 | 0.203 | 0.171 |
| 0.292 | 0.361 | 0.312 | 0.401 | 0.438 | 0.215 | 0.218 | 0.403 | 0.362 | 0.296 | 0.284 | 0.392 | 0.24 | 0.213 |
| 0.174 | 0.159 | 0.235 | 0.213 | 0.263 | 0.201 | 0.181 | 0.266 | 0.147 | 0.301 | 0.165 | 0.436 | 0.165 | 0.178 |
| 0.201 | 0.222 | 0.266 | 0.335 | 0.394 | 0.185 | 0.213 | 0.321 | 0.309 | 0.357 | 0.303 | 0.282 | 0.246 | 0.184 |
| 0.285 | 0.255 | 0.177 | 0.211 | 0.187 | 0.274 | 0.292 | 0.255 | 0.309 | 0.267 | 0.206 | 0.268 | 0.308 | 0.239 |
| 0.262 | 0.332 | 0.225 | 0.261 | 0.26 | 0.126 | 0.195 | 0.279 | 0.232 | 0.311 | 0.18 | 0.431 | 0.165 | 0.132 |
| 0.188 | 0.196 | 0.191 | 0.186 | 0.207 | 0.143 | 0.177 | 0.293 | 0.173 | 0.256 | 0.09 | 0.319 | 0.148 | 0.095 |
| 0.319 | 0.358 | 0.265 | 0.271 | 0.286 | 0.206 | 0.245 | 0.249 | 0.237 | 0.319 | 0.364 | 0.439 | 0.272 | 0.215 |
| 0.243 | 0.265 | 0.266 | 0.266 | 0.282 | 0.177 | 0.199 | 0.283 | 0.263 | 0.313 | 0.176 | 0.431 | 0.192 | 0.166 |
| 0.233 | 0.239 | 0.195 | 0.226 | 0.269 | 0.134 | 0.145 | 0.255 | 0.255 | 0.29 | 0.141 | 0.41 | 0.118 | 0.108 |
| 0.198 | 0.203 | 0.121 | 0.107 | 0.148 | 0.079 | 0.131 | 0.255 | 0.129 | 0.2719 | 0.109 | 0.391 | 0.093 | 0.078 |
| 0.399 | 0.374 | 0.276 | 0.261 | 0.297 | 0.264 | 0.322 | 0.291 | 0.292 | 0.409 | 0.405 | 0.547 | 0.33 | 0.275 |
| 0.305 | 0.256 | 0.171 | 0.214 | 0.215 | 0.172 | 0.229 | 0.469 | 0.298 | 0.342 | 0.065 | 0.457 | 0.201 | 0.157 |
| 0.227 | 0.249 | 0.157 | 0.125 | 0.107 | 0.131 | 0.117 | 0.215 | 0.179 | 0.255 | 0.152 | 0.419 | 0.132 | 0.135 |
| 0.372 | 0.397 | 0.344 | 0.513 | 0.466 | 0.428 | 0.377 | 0.536 | 0.389 | 0.579 | 0.283 | 0.54 | 0.361 | 0.376 |
| 0.001 | 0.006 | 0.081 | 0.165 | 0.136 | 0.018 | 0.067 | 0.261 | 0.096 | 0.209 | 0.152 | 0.298 | 0.016 | 0.002 |
|  | 0.005 | 0.049 | 0.2315 | 0.176 | 0.049 | 0.052 | 0.301 | 0.089 | 0.179 | 0.209 | 0.242 | 0.013 | 0.001 |
| **0.005** |  | 0.034 | 0.111 | 0.109 | 0.104 | 0.086 | 0.356 | 0.117 | 0.174 | 0.163 | 0.238 | 0.041 | 0.018 |
| 0.019 | **0.021** |  | 0.172 | 0.109 | 0.09 | 0.096 | 0.384 | 0.132 | 0.225 | 0.096 | 0.302 | 0.122 | 0.053 |
| 0.058 | 0.041 | 0.053 |  | 0.032 | 0.174 | 0.249 | 0.231 | 0.105 | 0.327 | 0.21 | 0.366 | 0.174 | 0.181 |
| 0.05 | 0.039 | 0.039 | **0.017** |  | 0.117 | 0.228 | 0.289 | 0.144 | 0.245 | 0.099 | 0.342 | 0.164 | 0.085 |
| **0.016** | 0.023 | **0.023** | 0.048 | 0.035 |  | 0.011 | 0.274 | 0.088 | 0.222 | 0.129 | 0.281 | 0.001 | 0.001 |
| 0.021 | 0.031 | 0.026 | 0.061 | 0.057 | **0.007** |  | 0.339 | 0.107 | 0.248 | 0.129 | 0.305 | 0.001 | 0.002 |

| 0.101 | 0.137 | 0.109 | 0.112 | 0.103 | 0.093 | 0.112 |  | 0.42 | 0.379 | 0.295 | 0.331 | 0.283 | 0.243 |
| --- | --- | --- | --- | --- | --- | --- | --- | --- | --- | --- | --- | --- | --- |
| 0.048 | **0.05** | 0.051 | 0.056 | 0.051 | 0.044 | 0.051 | 0.137 |  | 0.21 | 0.207 | 0.356 | 0.099 | 0.093 |
| 0.073 | 0.073 | 0.087 | 0.084 | 0.073 | 0.068 | 0.082 | 0.148 | **0.075** |  | 0.255 | 0.069 | 0.233 | 0.157 |
| 0.044 | 0.042 | 0.03 | 0.053 | 0.035 | 0.035 | 0.041 | 0.097 | **0.057** | **0.071** |  | 0.262 | 0.134 | 0.067 |
| 0.081 | 0.088 | 0.094 | 0.103 | 0.089 | 0.077 | 0.092 | 0.154 | **0.093** | **0.026** | **0.068** |  | 0.308 | 0.264 |
| **0.015** | **0.022** | **0.025** | 0.045 | 0.039 | **0.001** | **0** | 0.101 | 0.046 | **0.069** | 0.034 | 0.086 |  | 0.001 |
| **0.005** | **0.015** | **0.019** | 0.048 | 0.032 | **0.001** | **0.003** | 0.094 | 0.049 | 0.06 | 0.028 | 0.079 | **0.001** |  |
